# Detection of Herb-Symptom Associations from Traditional Chinese Medicine Clinical Data

**DOI:** 10.1155/2015/270450

**Published:** 2015-01-11

**Authors:** Yu-Bing Li, Xue-Zhong Zhou, Run-Shun Zhang, Ying-Hui Wang, Yonghong Peng, Jing-Qing Hu, Qi Xie, Yan-Xing Xue, Li-Li Xu, Xiao-Fang Liu, Bao-Yan Liu

**Affiliations:** ^1^School of Computer and Information Technology and Beijing Key Lab of Traffic Data Analysis and Mining, Beijing Jiaotong University, Beijing 100044, China; ^2^Guanganmen Hospital, China Academy of Chinese Medicine Sciences, Beijing 100053, China; ^3^School of Computing, Informatics and Media, University of Bradford, Bradford BD7 1DP, UK; ^4^China Academy of Chinese Medicine Sciences, Beijing 100700, China; ^5^Institute of Basic Theory of Traditional Chinese Medicine, China Academy of Chinese Medicine Sciences, Beijing 100700, China; ^6^Dongfang Hospital, Beijing 100078, China

## Abstract

*Background*. Traditional Chinese medicine (TCM) is an individualized medicine by observing the symptoms and signs (symptoms in brief) of patients. We aim to extract the meaningful herb-symptom relationships from large scale TCM clinical data.* Methods*. To investigate the correlations between symptoms and herbs held for patients, we use four clinical data sets collected from TCM outpatient clinical settings and calculate the similarities between patient pairs in terms of the herb constituents of their prescriptions and their manifesting symptoms by cosine measure. To address the large-scale multiple testing problems for the detection of herb-symptom associations and the dependence between herbs involving similar efficacies, we propose a network-based correlation analysis (NetCorrA) method to detect the herb-symptom associations.* Results*. The results show that there are strong positive correlations between symptom similarity and herb similarity, which indicates that herb-symptom correspondence is a clinical principle adhered to by most TCM physicians. Furthermore, the NetCorrA method obtains meaningful herb-symptom associations and performs better than the chi-square correlation method by filtering the false positive associations.* Conclusions*. Symptoms play significant roles for the prescriptions of herb treatment. The herb-symptom correspondence principle indicates that clinical phenotypic targets (i.e., symptoms) of herbs exist and would be valuable for further investigations.

## 1. Introduction

Traditional Chinese medicine (TCM) has been gradually developed from long-term clinical practices. Comprehensive data analysis about four diagnostic methods and long-term experiences is one of the main knowledge distilling approaches of TCM physicians. As an individualized diagnosis and treatment approach, the correspondence between personalized symptoms of patients and herbs prescribed constitutes basic elements of personalized treatment. The herb-symptom relationship (in which herbs are prescribed for specific symptoms) is a significant component. Furthermore, deriving common and effective herb-symptom relationships from large-scale clinical data of highly experienced TCM physicians can encourage the development of novel clinical prescriptions and the detection of effective empirical TCM clinical therapies [1].

In recent years, data mining methods have been widely applied to TCM clinical data for various clinical knowledge discoveries like syndrome differentiation, herb combination regularity, and patient clustering [2]. It is a significant task in TCM research to establish a data driven TCM clinical medicine research model based on real-world practices [3]. Given that there exist rich empirical and theoretical knowledge underlying TCM data, it is hoped that data mining would help the extraction of reliable and novel knowledge from the collected data.

The complicated relationships between clinical phenotypes and complex intervention used in TCM clinical practices highlight the important network structural patterns hidden in TCM clinical data. In particular, in TCM clinical settings, formula-syndrome correspondence (Fang-Zheng Correspondence [4, 5]) and herb modifications based on personalized symptoms (we call it herb-symptom correspondence [6, 7]) are two well-recognized approaches for TCM individualized treatment. The formula-syndrome correspondence principle is well established in both clinical practices and theoretical research [8]. However, although attempts were made to apply data mining algorithms for the discovery of specific symptom-herb association knowledge from TCM data, such as medical literature and clinical data, there are no studies yet to evaluate the herb-symptom correspondence in large-scale real-world clinical data. Furthermore, due to the similar efficacies held by different herbs, the traditional analytical methods that only treat herb names as distinct variables would fail to detect true herb-symptom association knowledge when a substantial number of efficacy similar herbs are prescribed in clinical data. Therefore, to investigate the correspondence between symptoms and herbs and detect significant and meaningful clinical relationships between symptoms and herbs, we calculate the symptom similarity and herb similarity between each patient pairs and these two similarities are used to evaluate the correlation between symptoms and herbs in clinical data. Furthermore, based on a chi-square correlation, we proposed a network-based correlation analysis (NetCorrA) framework to extract the real positive symptom-herb relationships from large-scale clinical data. The similarities of herb efficacy are also considered in this framework to detect the herb-symptom associations, which otherwise would be neglected by the influence of similar herbs.

## 2. Related Work

During the past two decades, we witnessed many data mining applications and studies to help extract medical knowledge from large-scale clinical data sets [9]. In recent years, a clinical data warehouse platform [10] has been developed to integrate the real-world electronic medical record data to support the medical knowledge discovery and clinical decision-making. For the traditional medicine, data mining method is also utilized widely; Afendi et al. discussed the usage of KNApSAcK Family DB in metabolomics, explain mining techniques such as principal component analysis (PCA), partial least square regression (PLSR), and multiway model, and show their application on Indonesian blended herbal medicines (Jamu) as a case study [11]. Many studies have focused on the discovery of the herb combination patterns in clinical prescriptions and the underlying structures of symptoms manifested on clinical patients, which use the data mining methods like latent tree model [12], association rules, and multidimensional reduction method [13]. In traditional medicine, the relationship between herb and formula has been investigated; Afendi et al. explored the relationship between Indonesian herbal plants and the efficacy of jamu [14]. Furthermore, to explore the complicated interactions between symptoms and other related medical entities, Li et al. [15] identified that the relative associated density (RAD) method is effective for TCM clinical data analysis, particularly for analysis of relationships between symptoms in diagnosis and generation of compact and comprehensible symptom feature subsets. Zhuang et al. [6] applied a biclustering method to analyze the compatibility of herbs and herb-symptom modules from clinical data.

However, as an important component for individualized TCM therapies, herb-symptom correlation phenomena still need to be systematically explored. For the detection of herb-symptom correlations, better methods are needed to filter the background noise induced by complicated prior knowledge such as herb efficacy in real-world clinical data.

## 3. Methods

### 3.1. Patient Symptom Similarity and Herb Similarity

**Figure 1:**
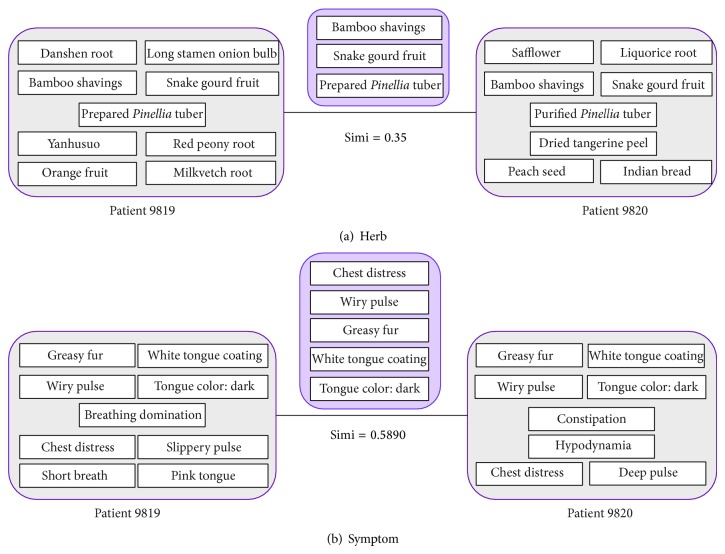
The principle of patient similarity procedure: (a) the procedure of patient symptom similarity; (b) the procedure of patient herb similarity.

For a data set, we assume that it has *n* herbs and *m* symptoms. As shown Figure 1, choosing a pair of patients, *P*_1_ and *P*_2_, the herb prescription used on patient *P*_1_ is defined as *H*_1_(*h*_11_, *h*_12_,…, *h*_1*n*_) and on *P*_2_ is *H*_2_(*h*_21_, *h*_22_,…, *h*_2*n*_); in the same way, symptom which is set on *P*_1_ is defined as *S*_1_(*s*_11_, *s*_12_,…, *s*_1*m*_) and on *P*_2_ is *S*_2_(*s*_21_, *s*_22_,…, *s*_2*m*_). If the herb *h*_*i*_ is contained in the herb prescription of patient *P*_1_, the *h*_*i*_ in the vector *H*_1_ is marked as 1; if not, the *h*_*i*_ in the vector *H*_1_ is marked as 0. This rule is also applied in the construction of vector *S*. Then, the herb similarity SimiH and symptom similarity SimiS of the patient pairs (*P*_1_ and *P*_2_) can be defined as:

(1) SimiH=cos⁡H1,H2=H1·H2H1·H2=∑h1i·h2i∑h1i2∑h2i2, 1≤i≤n,

(2) SimiS=cos⁡S1,S2=S1·S2S1·S2=∑s1j·s2j∑s1j2∑s2j2, 1≤j≤m

in which ‖*H*‖ represents the norm of vectors *H*. As we know, cosine similarity is a measure of similarity between two vectors of an inner product space that measures the cosine of the angle between them. Cosine similarity gives a useful measure of how similar two documents are likely to be in terms of their subject matter [16]. This means that if the patient pairs have more herbs in common, their SimiH would be closer to 1; likewise, if the patient pairs show more similar symptoms, their SimiS would be closer to 1.

After obtaining these two similarities between each patient pair, we calculate the correlation between symptoms and herbs by investigating the overlapping patterns of these two similarities between each patient pair. To compare the real-world data with random controls, we reshuffled each symptom and herb for patients to construct a random permutation coupled data set (Fisher-Yates shuffle method [17]). The correlation between symptoms and herbs is also calculated in this random controlled data set for comparison.

### 3.2. Chi-Square Test

We use the chi-square test (*χ* ^2^ test) method to calculate the relevance of herb-symptom relationship that appears in clinical cases (Table 1). A chi-square statistic is a measure of overall goodness of fit as well as a significance test of individual path coefficients [18]. The formula for chi-square test [19] is

(3) $$\begin{array}{c} \chi^{2}=\frac{{\left(ad-bc\right)}^{2}n}{\left(a+b\right)\left(c+d\right)\left(a+c\right)\left(b+d\right)} \end{array}$$

in which each of *a*, *b*, *c*, and *d* represents a real number in the fourfold table (Table 1), *n* represents the total number of cases, and *χ* ^2^ represents the chi-square statistics. The larger the value of *χ* ^2^ is, the stronger relevance the herb and the symptom hold.

Particularly, when the expected frequency is less than five and the total number of cases is greater than 40, we apply the following adjusted formula:

(4) $$\begin{array}{c} \chi^{2}=\frac{{\left(\left|ad-bc\right|-\left(n/2\right)\right)}^{2}n}{\left(a+b\right)\left(c+d\right)\left(a+c\right)\left(b+d\right)}. \end{array}$$

To discover highly significant, relevant, herb-symptom relationship knowledge, we chose those relationships with *P* values less than 0.05 as the reliable results. Furthermore, due to the large number of combinations of symptoms and herbs, the detection of herb-symptom associations is a large-scale multiple comparison problem, which needs to control the false discovery rate of associations. We use Bonferroni correction [20] to counteract this issue.

### 3.3. Network Extended Correlation Analysis

**Figure 2:**
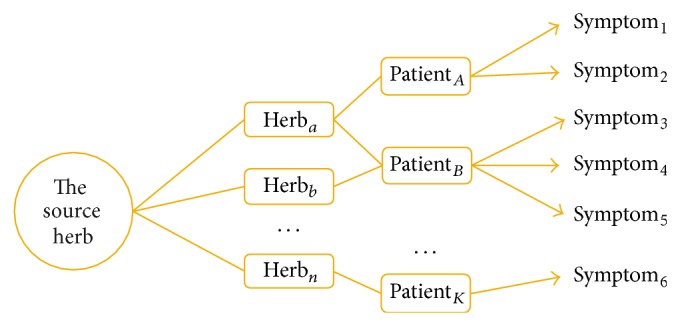
NetCorrA model; that is, NetCorrA: when we calculate the correlation between one herb and one symptom, we consider the neighbourhood of the herb (besides the herb itself) with significant shared efficacies as the expended herb set. We treated the expended herb set as the surrogate entity of herb to calculate the association between symptoms and herbs.

**Figure 3:**
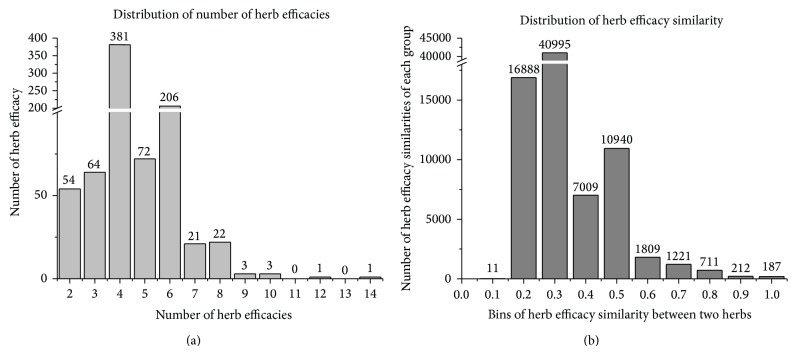
Distribution of number of herb efficacy and distribution of herb efficacy similarity: (a) the distribution of 373 herb efficacies of 829 herbs and (b) the distribution of the similarity of herb pairs with shared efficacies using cosine measure.

TCM herb is a complicated therapeutical entity holding various different ingredients and thus possesses different efficacies. This multiple efficacy property of herb leads to common efficacies held by different herbs. Therefore, in TCM clinical settings, physician could prescribe different herbs to treat patients with similar syndromes or symptoms. In this situation, the efficacy correlation between herbs would influence the detection of herb-symptom association patterns by classical correlation analysis methods like chi-square test. To address this issue of efficacy correlation between herbs, we propose a new correlation analysis method by incorporating the herb network with shared efficacies (Figure 2). In this method, when we calculate the correlation between one herb and one symptom, we consider the neighborhood of the herb (besides the herb itself) with significant shared efficacies as the expended herb set. We treated the expended herb set as the surrogate entity of herb to calculate the association between symptoms and herbs (Figure 2). This method is called “network-based correlation analysis method” (NetCorrA) to incorporate the efficacy similar herbs into the correlation analysis between herbs and symptoms. In NetCorrA, we consider the expedition of one herb to its neighborhood with significant overlapped efficacies; say three of the four distinct efficacies. Therefore, we evaluate the distribution of 373 herb efficacies of 829 herbs and calculate the similarity of herb pairs with shared efficacies using cosine measure. From the distribution of efficacies of herbs (Figure 3), we can see that the number of efficacy of herbs concentrates on [3, 6], and the number of herb efficacy similarity concentrates on [0.2,0.5]. However, when we evaluate the similarities between herbs with 3 more shared efficacies, it showed that most (73.58%) of the herb similarities are above 0.5; this means that half of the efficacies are the same in the herbs with no less than 3 common efficacies. Therefore, to be straightforward, we only extend the herb to its neighborhood, in which the herbs have no less than 3 efficacies in common with it.

## 4. Results

### 4.1. Clinical Data with Both Symptom Features and the Corresponding Herb Prescriptions

In our experiment, we used four clinical data sets: (1) the patient cases with liver-spleen disharmony, referred to as GPBT; (2) insomnia medical cases, referred to as INSOMNIA; (3) the clinical cases of children with Tourette's syndrome, referred to as TS; and (4) the inpatient cases with congestive heart failure, referred to as CHF. These data sets include symptoms and the related herb prescriptions as the two main feature sets.

As the data comes from clinical treatment and is highly noisy, the data preprocessing is needed. Firstly, all data is put in an extraction-transformation-loading (ETL) tool which is called medical integrator (MI) for clinical data integration, data cleaning, and preprocessing [10]. Based on this, we further ruled the herb name and symptom name artificially. Finally, clinical staff conducted validation to make sure of the data quality.

The basic information of the four data sets (the number of species of herb/symptom, number of patient, etc.) is depicted in Table 2.

Both the GPBT and INSOMNIA data sets are derived from an established clinical data warehouse [10] that has collected data since 2007. They come from practical formulae issued by several highly experienced TCM physicians and they can reflect the physicians' clinical experience. The data of TS comes from highly experienced TCM physicians in Dongfang Hospital, the Second Clinical Medical College of Beijing University of Chinese Medicine (BUCM). Seven hundred initial diagnosis records from 2005 to 2010 were collected in the outpatient treatment of children with TS. The CHF data comes from the Cardiovascularology Division of the First Teaching Hospital of Tianjin University of TCM, covering November 2011 to March 2013: the data has 253 cases. To filter the background noise in the data, we also reshuffled the original data sets to get their random coupled data set for comparison.

### 4.2. Herb-Symptom Correspondence Phenomenon

**Figure 4:**
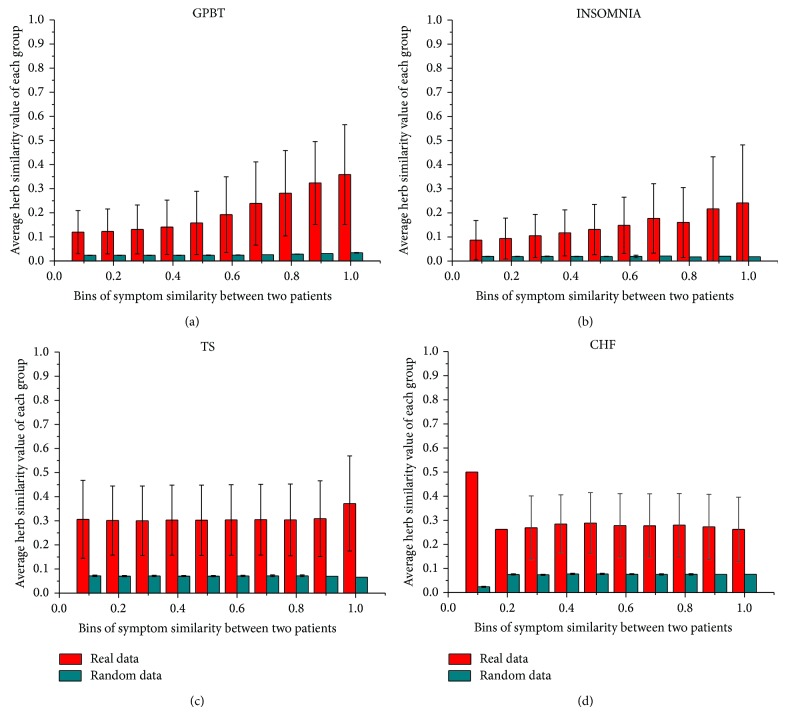
Correlation between herb and symptom of GPBT/INSOMNIA/TS/CHF: the *x*-axis represents “Bins of symptom similarity between two patients,” the *y*-axis represents “Average herb similarity value of each group.” The red column shows the result of real clinical data while the blue column shows the result of random data. The standard deviation of two types of data also shows in the figure. The correlation between herbs and symptoms presents a strong positive correlation, especially in data of GPBT and INSOMNIA.

**Figure 5:**
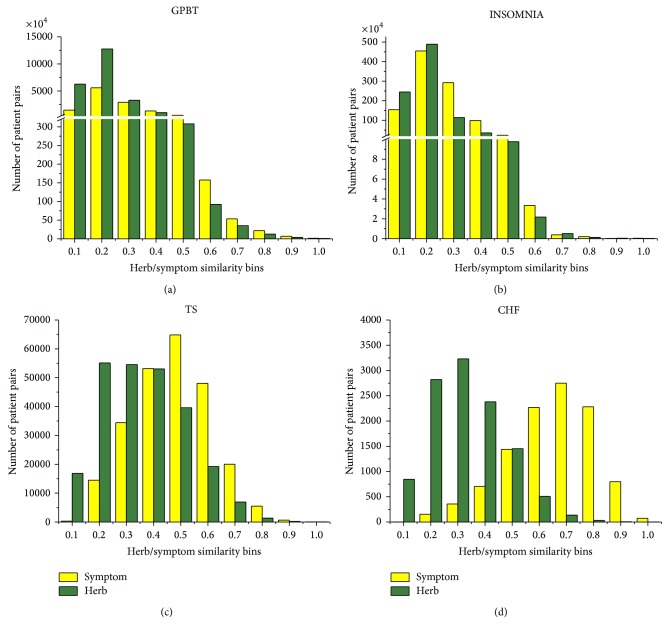
Distribution of symptom similarity and herb similarity of GPBT/INSOMNIA/TS/CHF: the *x*-axis represents “Herb/symptom similarity bins,” and *y*-axis represents “Number of patient-pairs.” The yellow column shows the symptom similarity data, while green column shows the herb similarity data. Most of the patient cases in GPBT and INSOMNIA data sets are in low symptom similarity and herb similarity (0.2 is the similarity in most cases), while the other two data sets both have much higher symptom and herb similarities (0.3 is the similarity in most cases for herbs and 0.5 or 0.7 is the similarities for symptom). Furthermore, there are clear disparities between herb similarity distribution and symptom similarity distribution in the latter two data sets.

Using ten bins of similarity between patient pairs and by calculating the overlap between patient pairs with both herb similarities and symptom similarities, we have an evaluation of the correlations between herbs and symptoms in the clinical data.  Figure 4 depicts the correlations of four data sets, in which the red column shows the result of the real data set while the blue column shows the random data set. We can clearly witness that the correlation between herbs and symptoms presents a strong positive correlation in TCM clinical treatment, especially in data of GPBT and INSOMNIA, which are from the clinical cases of several highly experienced physicians. Moreover, the Pearson coefficient of the correlation of GPBT approximates to 0.960 (the *P* value is 1.06*e* − 05) and INSOMNIA approximates to 0.964 (the *P* value is 7.03*e* − 06). This means that herbs and symptoms show a strong positive correlation in these two data sets. From the basic information of these two data sets (Table 2), we know that the patients in these two clinical case groups have rather diverse clinical manifestations (with thousands of symptom features) and various kinds of herb prescriptions (over 600 distinct herbs prescribed). Thus, in these kinds of typical personalized TCM clinical cases, the herb-symptom correspondence phenomenon (in other words, herb modification according to symptoms) is well established. However, the results of TS and CHF do not show a linear correlation (the Pearson coefficient of the correlation of TS is 0.047 and the *P* value is 0.250; the Pearson coefficient of the correlation of CHF is −0.047 and the *P* value is 0.904) although all the herb similarities between patient cases are much higher than the coupled random cases (0.3 versus less than 0.1 in TS and 0.25 versus less than 0.1 in CHF). This may be partly due to the much lower number of distinct symptoms recorded in these data sets (45 symptoms in TS and 29 symptoms in CHF). To further explore the causes of this difference held between these two conditions, we investigate the similarity distributions of these four data sets (in Figure 5). Figure 5 shows that most of the patient cases in GPBT and INSOMNIA data sets are in low symptom similarity and herb similarity (0.2 is the similarity in most cases), while the other two data sets both have much higher symptom and herb similarities (0.3 is the similarity in most cases for herbs and 0.5 or 0.7 is the similarities for symptom). Furthermore, there are clear disparities between herb similarity distribution and symptom similarity distribution in the latter two data sets. Therefore, we could conclude that the unusual symptom similar patients in the latter two data sets conceal the herb-symptom correspondence principle in the real-world clinical settings. There may exist other factors, such as common syndromes or disease categories that would correspond to the prescribed herb treatment. We further analyzed the core herb combinations prescribed in TS and CHF data sets and found that the clinical treatment of these two disease cases is actually based on two evident core formulae, which consist of rather fixed herbs. This means that there exists formula-disease correspondence other than herb-symptom correspondence in these two typical cases.

### 4.3. Detection of the Herb-Symptom Association Knowledge

To obtain the significant herb-symptom relationships, we extracted the herb-symptom relationships whose *P* values were less than 0.05 from the chi-square test and NetCorrA method (Table 3). To evaluate the quality of the relationships in terms of clinical coherence, we selected 1000 herb-symptom records of GPBT and INSOMNIA, respectively, and let TCM clinical experts manually label whether there exists correlation or not (correlation: 1; no correlation: 0). The top 50 most-used herbs in the GPBT/INSOMNIA data set are selected. For each selected herb, we chose 20 herb-symptom records which own minimum chi-square values.  Table 4 lists the specific herb-symptom relationship results whose clinical label is 1. Clinical label marked 1 means that the herb and the symptom exist in correlation with the clinical treatment. We found that the consistency between correlation analysis and the labels of medical experts had acceptable accuracy. It had 70% of the chi-square test and 72.5% of NetCorrA in GPBT and 71.8% of the chi-square test and 73.2% of NetCorrA in INSOMNIA. In particular, the NetCorrA rectified many false positive herb-symptom correlations, which were detected by the chi-square test as significant correlations but were labeled as no correlations.  Table 5 lists specific herb-symptom relationship results whose clinical label is 0. For example, in the GPBT data results, the *P* value of Chinese angelica-red throat association in NetCorrA was 0.557, which showed no significant correlation, while the *P* value was 0 in the chi-square test model, which means that there is a very high correlation. In addition, in INSOMNIA data results, the *P* value of fresh* Rehmannia*-tongue score's association in the NetCorrA herb model was 0.221, which is 4 × 10^6^ times more than that in the chi-square test. These two associations have the clinical labels of 0, which means that there is no relationship between the herbs and the symptoms. Therefore, the common-effect herb model does filter out the negative symptom-herb relationships. These significant herb-symptom relationships (Table 3), summarized from a large scale of real clinical data, are reliable and are meaningful for TCM clinical diagnosis and treatment.

## 5. Discussion

It is well known that syndrome is the main diagnosis of TCM and it is the target of herb precription as well [21, 22]. This principle is also held by most traditional medicines like Kampo diagnosis [23]. This means that the patients that have different syndromes would have much different herb prescriptions as treatment even though they have similar symptoms [24]. Meanwhile, in TCM formula theories, herbs are mainly described by efficacies and herb properties like “hot” and “cold,” which are different from symptom oriented indications [25].

In this paper, we investigate and detect the correlation between symptoms and herbs by calculating the similarities bewteen each patient pairs. Four data sets derived from real-world clinical practices are used to investigate the general symptom-herb correlation phenomena and discover specific regularities between symptoms and herbs. The results indicate that there exists strong correlation between symptoms and herbs in clinical records, particularly, in the outpatient cases treated by highly experienced TCM physicians. Therefore, the result confirms that “symptomatic treatment” is one of the basic principles adhered to by TCM physicians during the prescription of individualized clinical treatment. This “herb-symptom correspondence” principle is acutally hidden in the real-world clinical data. In other words, the actual data suggests that TCM physicians make herb additive or subtractive prescriptions according to the patient's symptoms in clinical treatment particularly for patients with clinical manifestations. Specifically, from the result of the GPBT syndrome data set, it shows strong positive correlation between herb-similarity and symptom-similarity (Pearson coefficient = 0.96). This could be used to further investigate the clinical phenotype targets of herbs or herb prescriptions that may phenotype screening for novel drug development.

Furthermore, based on the verified herb-symptom corresponding principle, we demonstrate the approach to obtain the significant herb-symptom relationships by chi-square test and NetCorrA methods. The results showed that NetCorrA performs better than chi-square test to detect the true herb-symptom associations; in particular, it filters many false positive associations, which otherwise would be detected by chi-square test. As we know that the underlying mechanisms of clinical manifestations like symptoms are not elucidated yet and the efficacies of herbs are not fully investigated, due to the complicated and various manifestations of patients in clinical settings, the herb prescriptions produced by TCM physicians contain many novel empirical skills that are not covered by existing classical knowledge, which would provide valuable resources for clinical data mining and medical research. Therefore, these results contain some specific knowledge held in the mind of TCM physicians, which would play a significant role in the distilling and refinement of empirical knowledge of TCM physicians. In addition, young TCM physicians could use these specific symptom-herb associations for references while they are conducting regular clinical tasks.

However, because herb prescription is a kind of combination therapy, which often includes 10–20 different herbs as a whole for disease treatment, the reliable detection of herb-symptom associations needs further consideration of the interaction between herbs in prescriptions. Currently, in this paper, NetCorrA has not incorporated herb combinations as factors to detect the herb-symptom associations, which can further be investigated by using the methods like gene set enrichment analysis approach [26]. Another limitation of the current work is that we do not evaluate the herb-symptom associations as effective or not because we have not included the outcome related information like symptom disappearance in the EMR data. This would be investigated in our future work.

Being one kind of the key clinical manifestations of patients, symptoms play significant role for clinical diagnosis and treatment, which evidently have their underlying molecular mechanisms [27]. Therefore, the detection of herb-symptom associations and further of the herb-symptom interaction would be useful for investigation of the underlying molecular mechanisms of traditional herb medicines [28], which may be helpful to change traditional medicine from empirical medicine to evidence-based and molecule-oriented medicine.

## Figures and Tables

**Table 1:** Chi-square test sata.

	Greasy fur (Occurred: 1)	Greasy fur (Not occurred: 0)
Danshen root (used: 1)	933 (*a*)	4192 (*b*)
Danshen root (not used: 0)	2221 (*c*)	13925 (*d*)

**Table 2:** Comparison of four data sets.

Data set	Feature types	Number of features	Number of patient cases	Average number of herbs in the formula/of symptom	Common number of patient cases
GPBT	Herb	624	21345	15	21271
	Symptom	6487	23082	8	
INSOMNIA	Herb	618	4537	14	4533
	Symptom	1977	4558	11	
TS	Herb	162	699	11	699
	Symptom	45	700	9	
CHF	Herb	194	148	12	148
	Symptom	29	249	13	

**Table 3:** Summary of herb-symptom relationship discovery.

MODELS	ITEMS	GPBT	INSOMNIA	TS	CHF
Chi-square test	Number of related herbs	624	618	189	194
	Number of herb-symptom results (*P* values < 0.05)	60,249	20,953	213	60
	Number of herb-symptom results (*q*-values < 0.05)	44,271	11,867	23	0

NetCorrA	Number of related herbs	366	280	85	89
	Number of herb-symptom results (*P* values < 0.05)	38,463	12,923	178	75
	Number of herb-symptom results (*q*-values < 0.05)	28,404	7,169	3	0

**Table:** (a) GPBT herb-symptom relationship

	Herb	Symptom	Clinical label (correlation: 1)	Chi-square test: *P* value	Chi-square test: *q*-value	NetCorrA: *P* value	NetCorrA: *q*-value
1	Chinese angelica	Hypogastralgia	1	0	0	0.0004	0.0032
2	Chinese angelica	Greasy fur	1	0	0	2.44*E* − 05	0.0002
3	Chinese angelica	Cough	1	0	0	1.03*E* − 12	2.63*E* − 11
4	Danshen root	Dark tongue	1	0	0	0.0065	0.0342
5	Danshen root	Cough	1	0	0	6.66*E* − 16	2.23*E* − 14
6	Danshen root	Anorexia	1	0	0	0	0
7	Liquorice root	Relaxed pulse	1	0	0	2.24*E* − 14	6.65*E* − 13
8	Liquorice root	Angina	1	0	0	0.0006	0.0043
9	Baical skullcap root	Fever	1	0	0	4.25*E* − 06	4.76*E* − 05
10	Baical skullcap root	Yellow fur	1	0	0	2.96*E* − 06	3.42*E* − 05

**Table:** (b) INSOMNIA herb-symptom relationship

	Herb	Symptom	Clinical label (correlation: 1)	Chi-square test: *P* value	Chi-square test: *q*-value	NetCorrA: *P* value	NetCorrA: *q*-value
1	Fresh *Rehmannia *	Red tongue	1	0	0	0	0
2	Fresh *Rehmannia *	Distracted	1	7.82*E* − 10	4.51*E* − 08	1.03*E* − 11	8.28*E* − 10
3	Fresh *Rehmannia *	Dried manure	1	3.59*E* − 10	2.16*E* − 08	2.06*E* − 07	7.58*E* − 06
4	Poria with hostwood	Palpitation	1	1.22*E* − 05	0.0003	3.72*E* − 11	2.77*E* − 09
5	Poria with hostwood	Yellow fur	1	2.52*E* − 06	7.26*E* − 05	1.00*E* − 04	0.0019
6	Poria with hostwood	Red tongue	1	8.39*E* − 07	2.67*E* − 05	1.37*E* − 09	8.06*E* − 08
7	Long Gu	Dizziness	1	1.12*E* − 10	7.35*E* − 09	0	0
8	Long Gu	Palpitation	1	2.27*E* − 11	1.65*E* − 09	2.20*E* − 05	0.0005
9	Long Gu	Dreaming often	1	4.94*E* − 12	3.96*E* − 10	0.0029	0.0304
10	Long Gu	Dizziness	1	2.41*E* − 09	1.26*E* − 07	1.07*E* − 13	1.19*E* − 11

**Table:** (a) GPBT herb-symptom relationship

	Herb	Symptom	Clinical label (no correlation: 0)	Chi-square test: *P* value	Chi-square test: *q*-value	NetCorrA: *P* value	NetCorrA: *q*-value
1	Chinese angelica	White tongue coating	0	0	0	0.2685	0.5993
2	Chinese angelica	Red throat	0	0	0	0.5573	0.9024
3	Chinese angelica	Thin fur	0	0	0	0.2679	0.5983
4	Danshen root	Red throat	0	0	0	0.9232	0.9988
5	Danshen root	White tongue coating	0	0	0	0.1605	0.4297
6	Danshen root	Slippery pulse	0	0	0	0.2459	0.5689
7	Liquorice root	Red throat	0	0	0	0.2346	0.5531
8	Liquorice root	Belch	0	0	0	0.1919	0.4841
9	Baical skullcap root	Little phlegm	0	0	0	0.8659	0.9953
10	Baical skullcap root	White tongue coating	0	0	0	0.3811	0.7394

**Table:** (b) INSOMNIA herb-symptom relationship

	Herb	Symptom	Clinical label (no correlation: 0)	Chi-square test: *P* value	Chi-square test: *q*-value	NetCorrA: *P* value	NetCorrA: *q*-value
1	Chinese angelica	Oliguria	0	1.07*E* − 08	4.99*E* − 07	0.2352	0.5299
2	Chinese angelica	Deep pulse	0	7.07*E* − 09	3.41*E* − 07	0.1422	0.4053
3	Chinese angelica	Hypodynamia	0	6.32*E* − 09	3.07*E* − 07	0.4162	0.6660
4	Chinese angelica	Pale white tongue	0	3.02*E* − 13	2.76*E* − 11	0.1203	0.3657
5	Poria with hostwood	Rootless fur	0	1.55*E* − 05	3.67*E* − 04	0.0876	0.3004
6	Poria with hostwood	Thin fur	0	3.42*E* − 09	1.75*E* − 07	0.1548	0.4259
7	Poria with hostwood	Anorexia	0	1.33*E* − 07	5.10*E* − 06	0.0304	0.1476
8	Ginseng	Expectoration	0	8.72*E* − 07	2.77*E* − 05	0.3982	0.6562
9	Long Gu	Night sweat	0	9.97*E* − 06	2.49*E* − 04	0.7702	0.7868
10	Long Gu	Lumbago and knee arthralgia	0	3.11*E* − 06	8.72*E* − 05	0.4869	0.6999

**Table:** (c) TS herb-symptom relationship

	Herb	Symptom	Chi-square test: *P* value	Chi-square test: *q*-value	NetCorrA: *P* value	NetCorrA: *q*-value
1	Figwort root	Feverishness in palms and soles	0.0077	0.3001	0.1121	0.6405
2	Figwort root	Head flick	0.0271	0.6168	0.2739	0.8109
3	Figwort root	Abnormal tongue fur	0.1199	0.9953	0.0057	0.2178
4	Figwort root	Kengkeng	0.1435	0.9953	0.1163	0.6479
5	Tangshen	Malnutrition	0.0252	0.6169	0.0290	0.3843

**Table:** (d) CHF herb-symptom relationship

	Herb	Symptom	Chi-square test: *P* value	Chi-square test: *q*-value	NetCorrA: *P* value	NetCorrA: *q*-value
1	Tangshen	Eye mental deficiency	0.0018	0.9946	0.0291	0.9954
2	Tangshen	Wheezing	0.0033	0.9946	0.0723	0.9988
3	Tangshen	Mental burnout	0.0444	0.9946	0.2704	0.9985
4	Cassia twig	Burnout	0.1127	0.9946	0.0549	0.9825
5	Cassia twig	Short breath	0.0092	0.9946	0.0019	0.8759
